# Exploring the Relationship Between Knowledge, Attitudes, Self-Efficacy, and Infection Control Practices Among Saudi Arabian Nurses: A Multi-Center Study

**DOI:** 10.3390/healthcare13030238

**Published:** 2025-01-24

**Authors:** Amal Alsulami, Lailani Sacgaca, Petelyne Pangket, Eddieson Pasay-an, Fatmah Ahmed Al Amoudi, Maha Sanat Alreshidi, Nojoud Alrashedi, Romeo Mostoles, Joyce Buta, Benito Areola, Allen Joshua Dominguez, Analita Gonzales

**Affiliations:** 1 Department of Community and Mental Health Nursing Sciences, College of Nursing, Taif University, Taif City 21944, Saudi Arabia; turabea20132014@gmail.com; 2 Department of Nursing, Prince Sultan Military College of Health Sciences, Al Amal Dhahran City 34313, Saudi Arabia; lailani@psmchs.edu.sa (L.S.); fatimaamodi@psmchs.edu.sa (F.A.A.A.); 3 College of Nursing, King Khalid University, Abha 61481, Saudi Arabia; edpasayan@gmail.com; 4 College of Nursing, University of Hail, Hail City 2440, Saudi Arabia; maha-838@hotmail.com (M.S.A.); nojodsss333@hotmail.com (N.A.); rpmostolesjr@gmail.com (R.M.J.); j.buta@uoh.edu.sa (J.B.); 5 College of Nursing, Shaqra University, Riyadh City 11961, Saudi Arabia; areola@su.edu.sa (B.A.J.); adominguez@su.edu.sa (A.J.D.); 6 Nursing Administration and Education Department, Faculty of Nursing, University of Tabuk, Tabuk 47512, Saudi Arabia; a_gonzales@ut.edu.sa

**Keywords:** self-efficacy, knowledge, attitude, standard precaution, nurses, compliance

## Abstract

**Introduction:** Healthcare-associated infections are a significant risk to patients and the healthcare system. Differences in knowledge, attitudes, and practices among nurses have limited standardized precautions. Improving self-efficacy can enhance compliance with standard precautions, protect patients, and preserve the credibility of healthcare institutions. **Aim:** This study aimed to determine the mediating effects of self-efficacy on nurses’ knowledge, attitude, and compliance with standard precautions in Saudi Arabia. **Methods:** This cross-sectional study was conducted in four hospitals in Dharan, Saudi Arabia, between July and October 2023. The respondents were selected through a multistage sampling of 305 nurses from wards and special areas. **Results:** participants demonstrated a moderate level of knowledge (M = 19.00, SD = 2.17), positive attitudes (M = 55.86, SD = 10.22), intermediate compliance (M = 72.44, SD = 11.47), and moderate self-efficacy (M = 29.99, SD = 11.40). Respondents who possessed more knowledge demonstrated positive attitudes toward standard precautions (r = 0.256, *p* < 0.001) and were more likely to comply (r = 0.376, *p* < 0.001). Higher self-efficacy levels were significantly positively correlated with knowledge (r = 0.391, *p* < 0.001), attitude (r = 0.311, *p* < 0.001), and compliance (r = 0.385, *p* < 0.001). The direct effect of knowledge on compliance was estimated to be 0.115 (*p* < 0.001), while the direct effect of attitude was slightly weaker (0.014, *p* = 0.049). Self-efficacy indirectly increased compliance through its influence on knowledge and attitude, with indirect effects of 0.039 (*p* < 0.001) and 0.008 (*p* = 0.002), respectively. **Conclusions:** Nurses with higher knowledge of infection control and positive attitudes toward established protocols were more likely to comply consistently. This association is further strengthened by self-efficacy. This confidence enhanced their understanding of the specific procedures, reinforced their positive beliefs about the importance of protocols, and ultimately led to greater adherence.

## 1. Introduction

Healthcare-associated infections (HAIs) are a serious hazard for patients and the healthcare environment globally [1], just as they are in Saudi Arabia. A recent study found that almost a quarter (23%) of ICU infections in Saudi hospitals were HAIs and, consequently, increased morbidity as well as health costs [2]. These infections pose a significant threat and require robust infection prevention and control (IPC) policies [3,4]. Despite limited research on IPC practices in Saudi Arabia, nationally representative data on clinical and healthcare-associated infection rates and related factors are scarce [5]. This lack of robust data not only obscures the true extent of the HAI problem but also hinders the formulation of evidence-based policies to improve IPC practices.

While previous research has highlighted the importance of knowledge and attitudes in influencing IPC adherence [6], the role of self-efficacy in this context remains understudied in Saudi Arabia. Self-efficacy is an individual’s belief in their ability to successfully perform specific actions required for a given task [7]. To others, self-efficacy is the opinion about the self in performing certain behaviors necessary for accomplishing a task or achieving certain objectives [5]. As such, self-efficacy greatly reinforces the compliance of health personnel staff with IPC guidelines [8]. For example, a study from Saudi Arabia showed that even though healthcare providers appreciated the need for IPC, their self-efficacy prevented them from effectively implementing these practices [5]. This implies that if self-efficacy is improved among the health care workers, then adherence to IPC measures can also be enhanced, which is significant in reducing the occurrence rates of HAIs. Furthermore, the characteristics and culture within the Saudi healthcare environment determine the attitudes toward patient safety and IPC practices. Cultural aspects such as hierarchy and traditionalism could impact healthcare providers’ willingness to comply [9]. It has been observed that the development of a safety culture has a significant bearing on patient safety performance [10]. In Saudi Arabia, it is crucial to pursue a policy that promotes a safety culture among health providers and encourages them to continuously learn to raise adherence to IPC principles [11]. Also significant are the interrelations among knowledge, self-efficacy, and attitudes, as they help in understanding aspects of compliance behaviors where the moderating effects of moral beliefs on the self-efficacy compliance relationship are concerned [12] This multi-perspective approach to the problem aims to overcome the knowledge limitations of the healthcare workers that prevent adherence to the IPC protocols by enabling them [12].

This study is important to fill the gap in the current information on infection control measures practiced by nurses in Saudi Arabia. Understanding the relationship between self-efficacy, knowledge, and attitudes and their compliance with infection control measures to stop the transmission of infectious diseases will provide information that can be used in policy formulation and designing educational programs for the Saudi healthcare system. In this sense, comprehending the different problems nurses experience will bring about effective approaches that increase the IPC compliance rate. This, in turn, increases patient safety and more so decreases HAIs in healthcare as a whole [13,14]. Therefore, this study aims to investigate the correlation among self-efficacy, knowledge, attitude, and compliance in preventing the spread of contagious diseases among nurses in Saudi Arabia.

## 2. Methods

### 2.1. Research Design

This cross-sectional study explored the mediating effects of self-efficacy on nurses’ knowledge, attitudes, and compliance with standard precautions in Saudi Arabia.

#### 2.1.1. Settings

Dhahran Province from the Eastern region of Saudi Arabia was chosen as the setting for this study because of its dense population and variety of health services. Infection control measures are commonly practiced by nursing staff working at four representative hospitals located in the Dhahran Province. These are Pro-care hospitals, which are private tertiary healthcare centers with a 100-bed capacity, offering specialties in cardiology, oncology, and neurology; Saudi German Hospital, a 200-bed private multi-specialty, with focused areas in gynecology, pediatrics, and internal medicine; Dammam Medical Complex, a Ministry of Health-owned 400-bed public tertiary healthcare facility focusing on regional healthcare delivery; and Mouwasat Hospital, a private hospital with 200 beds, providing specialized health services including orthopedic, ophthalmologic, and dental services. All these hospitals are committed to the provision of quality healthcare services to the people of Dhahran but differ in the extent of services rendered, ownership, and the areas of focus.

#### 2.1.2. Participants

To determine the minimum sample size required for our study, we used the Raosoft online sample size calculator (http://www.raosoft.com/samplesize.html, accessed on 14 October 2024). This calculator employs the formula n = (Z^2^ * p(1 − p))/e^2^, where n represents the minimum sample size, Z is the Z-score for a 95% confidence interval (1.96), p signifies the estimated population proportion (0.02), and e represents the desired margin of error (5%) [15]. Based on the chosen 95% confidence level and the values entered for the population proportion and margin of error, the Raosoft calculator indicated a minimum sample size of 305 participants from the total population of 1500 from the four hospitals. Multistage sampling was performed using a representative sample. A total of 1500 healthcare workers were divided into four categories, each corresponding to the four participating hospitals. The available nurses in every hospital formed the basis upon which proportional quotas were determined using Raosoft software (v.2021). The sample sizes from each hospital corresponded to the overall healthcare labor force. From the various departments, namely, Nursing, Medicine, Surgery, and so forth, healthcare workers from the different departments in each selected hospital and a random sample were utilized. This study included registered nurses with at least one year of clinical experience who were currently employed at one of the participating hospitals. Nurses in managerial positions, outpatient departments, or on leave during the data-collection period were excluded. All participants provided voluntary informed consent.

#### 2.1.3. Data Gathering Tools

This study employed established and validated instruments from the original authors. The authors in this study maintained the original items and language, which were English in the questionnaire. The remainder of this paper is organized as follows. Section 1 delves into the respondents’ profiles, including the name of their current hospital and area of assignment. The subsequent sections refer to the four questionnaires used.

Section 2 examines the knowledge of HCWs on infection control standard precautions based on the work of Abalkhail et al. [16]. This questionnaire included 20-item dichotomous scales (1 = yes/0 = no) that assessed the respondents’ knowledge of standard precautions, focusing on hand hygiene, nosocomial infections, and standard precautions. The maximum possible score is 20 points, ranging from 0 to 20 points. The overall level of knowledge was classified as poor (<10 points, <50% correct answer), moderate (10–15 points, 50–79% correct answer), and good (16–20 points, 80–100% correct answers).

The attitude towards the standard precautions tool, rooted in Mohd-Nor and Bit-Lian [17], is evaluated in Section 3. It comprises 15 items and five Likert-scale questions (1 = strongly disagree, 5 = strongly agree) to measure participants’ attitudes toward standard precautions. The maximum possible score is 75 points, ranging from 15 to 75 points. Poor: <37 points (<50% score); Moderate: 37–55 points (50–79% score); Positive: 56–75 points (80–100% score) [16].

The Compliance Questionnaire [18] is assessed with 20-item questions. It uses five Likert-scale questions (0 = never, 4 = always) to evaluate nurses’ compliance with standard precautions. The maximum total score is 80 (out of 80). The score can be classified as low for scores below 50 percent, intermediate for those between 50 and 75 percent, and high for those above 75 percent [19].

The General Self-Efficacy (GSE) Questionnaire developed by Schwarzer and Jerusalem [20] is explained in Section 2. It contains four Likert-scale questions (ranging from 1 to 4, with 1 signifying “not at all true” and 4 signifying “exactly true”), designed to assess participants’ self-efficacy. The questionnaire consisted of ten items. All items were summed to achieve a total score. In the case of the GSE, the total score ranged from 10 to 40. The higher the score, the higher the perceived self-efficacy of the individual.

To ensure the instrument’s suitability within the specific context of this study and the target population, the authors subjected them to further validity with the three experts in the field (one psychometrician and two university researchers). The reliability testing in the current study was conducted with the 23 respondents. Cronbach’s alpha coefficients demonstrated good internal consistency for all instruments: 0.87 for knowledge, 0.85 for attitude, 0.84 for compliance, and 0.89 for GSE.

### 2.2. Data Gathering Procedure

After obtaining approval from the ethics committee, the study officially commenced. The initial collaboration involved securing permission from the directors of four hospitals to survey their nursing staff. After receiving authorization, the researchers and nursing offices identified eligible participants who met the inclusion criteria. This was followed by an orientation session, in which staff nurses were informed of the voluntary nature of their participation and that they could withdraw at any time. Finally, the nurses who agreed to participate were sent a Google Forms survey link containing an informed consent form via e-mail. Before opening Google Link, instructions were provided if participants had completed the questionnaire via Google Link, indicating their understanding of the study information and serving as their implied consent to participate.

### 2.3. Ethical Considerations

The ethics review committee of the Prince Sultan Military College of Health Sciences reviewed and approved this study (IRB-2024-NUR-35 dated 13 May 2023). Participation was voluntary, and withdrawal was possible at any time without consequences. The data were stored securely on a hard drive and were accessible only to researchers.

### 2.4. Data Analysis

For data analysis, we applied the Jeffreys’ Amazing Statistics Program (JASP) software version 0.18.0. The data files were cleaned and verified for correctness and missing data were addressed prior to performing the analysis properly. Through the use of multiple imputation techniques, and employing the chained equations (MICE) method, the missing data underwent multiple imputations in an attempt to reduce the bias that was said to have been contributed by missing data. The MICE technique is based on the principle of fully conditional specification, whereby each variable is imputed on the basis of other variables that are present in the dataset, and, thus, the relationships among the variables are retained [21].

The examined features were reported as proportions complemented by frequency distributions.

The descriptive statistics for continuous variables (self-efficacy, knowledge, attitude, compliance) were given as mean ± standard deviation and median (IQR-ed). The mean was chosen as the putative value of the expected value based on its normal distribution along with the standard deviation. Independent *t*-test and Analysis of Variance (ANOVA) were used to determine significant variations between different groups under the continuous variables. Pearson correlation analysis of corresponding variables was computed to investigate the linear correlation between self-efficacy, knowledge, attitude, and compliance with standard precaution measures. Indeed, Pearson’s correlation coefficient was the appropriate measure of the linear relationship between discrete variables. The correlation coefficients were subjected to a two-tailed test with a significance level set at 0.05. Working with knowledge, attitude, and self-efficacy as mediating variables between independent and dependent variables required a mediation analysis following a bootstrapping strategy. Bootstrapping qualifies the confidence intervals for indirect effects using data resampling through replacement [21].

## 3. Results

The majority of the participants were female (76.4%), with a smaller proportion being male (23.6%). A high percentage of participants (95.1%) attended training on standard precautions or infection control. The highest degree of nursing among the participants was a bachelor’s degree (52.8%), followed by a diploma (27.9%), a master’s degree (15.1%), and a doctorate degree (4.3%). The majority of the participants (60%) had between one and five years of work experience (Table 1).

The participants had moderate knowledge (19.00 ± 2.17), moderate attitude (55.86 ± 10.22), an intermediate level of compliance (72.44 ± 11.47), and moderate self-efficacy (29.99 ± 11.40) (Table 2).

Table 3 presents the significant differences based on several demographic factors. Regarding gender, females demonstrated significantly lower attitude scores compared to males (Female Mean: 53.6481, Male Mean: 63.0139, t = −6.0527, *p* < 0.0001), while no significant differences were observed in knowledge, compliance, or general self-efficacy. Training attendance significantly impacted knowledge (Attended Mean: 19.2276, Not Attended Mean: 14.6667, t = 2.9016, *p* = 0.0115), compliance (Attended Mean: 72.9310, Not Attended Mean: 62.9333, t = 2.3836, *p* = 0.0311), and general self-efficacy (Attended Mean: 30.5828, Not Attended Mean: 18.5333, t = 7.6679, *p* < 0.0001), with those who attended training demonstrating higher scores in all three areas. Regarding the highest degree obtained, professionals with Bachelor’s degrees exhibited significantly higher compliance rates (Bachelor’s Mean: 73.820, F = 7.7768, *p* < 0.0001), and those with Bachelor’s and Master’s degrees had significantly higher levels of general self-efficacy. Finally, professionals with 1–5 years of experience demonstrated significantly higher compliance rates (1–5 years Mean: 73.6995, F = 3.1277, *p* = 0.0261).

Knowledge showed a significantly positive correlation with attitude (r = 0.256, *p* < 0.001), indicating that higher knowledge levels are related to more positive attitudes. Compliance demonstrated significant positive correlations with knowledge (r = 0.376, *p* < 0.001) and attitude (r = 0.249, *p* < 0.001), suggesting that greater knowledge and more positive attitudes are associated with increased compliance. Self-efficacy was significantly positively correlated with knowledge (r = 0.391, *p* < 0.001), attitude (r = 0.311, *p* < 0.001), and compliance (r = 0.385, *p* < 0.001), indicating that higher levels of self-efficacy were linked to greater knowledge, more positive attitudes, and higher compliance (Table 4).

The path coefficients for the relationships between variables in the structural equation model are shown in Table 5. The data suggest that self-efficacy significantly predicts compliance in a strong and positive manner (path coefficient = 0.254, *p* < 0.001). Knowledge has an even greater direct and positive predictive effect on compliance (path coefficient = 0.115, *p* < 0.001). In addition, attitude has a positive but weak direct effect on compliance which is statistically significant (path coefficient = 0.014, *p* = 0.050). Last but not least, knowledge has a strong and positive predictive effect on self-efficacy (path coefficient = 0.154, *p* < 0.001), and self-efficacy is also positively predicted by attitude (path coefficient = 0.030, *p* < 0.001).

The R-squared value for the compliance model was 0.218. This means that 21.8% of the variance in compliance could be explained by the independent variables included in the model. The R-squared value for the self-efficacy model was 0.200. This indicates that 20% of the variance in self-efficacy could be attributed to the independent variables in the model.

**Figure 1:**
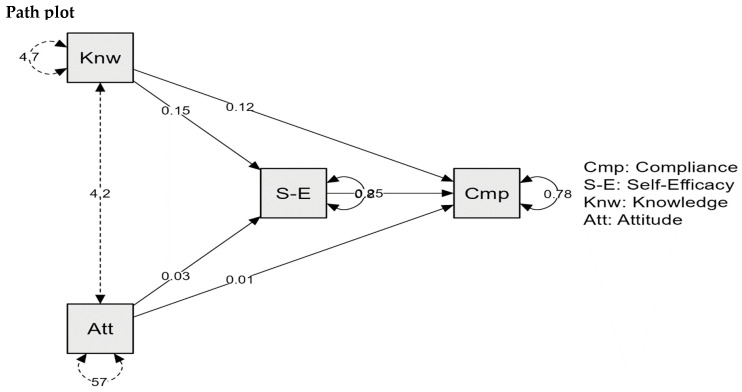
Mediation analysis model. Legend: Knw: Knowledge; Att: Attitude; S-E: Self-Efficacy; Cmp: Compliance.

To validate the research hypotheses and examine the construct relationships, JASP 0.18.0.0 was used. The path coefficient findings are presented in Figure 1.

Knowledge showed a significant positive path coefficient of 0.115 for compliance (*p* < 0.001), highlighting its direct influence on compliant behavior. Attitude had a marginally significant positive path coefficient of 0.014 for compliance (*p* = 0.050), suggesting a weaker direct influence than knowledge (Figure 1).

## 4. Discussion

This study aimed to determine the role of nurses’ self-efficacy in their knowledge, attitude, and compliance with standard precautions. The findings showed that the survey respondents possessed moderate levels of knowledge, attitudes, compliance, and self-efficacy associated with practices of infection control. This finding concurs with previous studies that reported a satisfactory level of knowledge and practices of infection control among health professionals while their attitudes towards infection control were neutral, indicating that gaps in knowledge and practice still exist [22,23]. Likewise, in a study focusing on self-efficacy, it was established that self-efficacy affects compliance with hand hygiene among nurses; thus, self-efficacy improvement could result in better compliance with required infection control procedures [24]. Attitudes towards infection control amongst the participants in this study are manageable, and, as such, there is an opportunity to facilitate improvement. There is evidence indicating a strong correlation between nurses’ attitudes and their self-efficacy to comply with infection prevention practices [24]. Such evidence highlights the need for training programs to have a dual purpose of providing information and changing attitudes for better adherence to infection control practices. It was stressed in an earlier study the importance of educational reinforcement to refresh the memory of the practicing health workers on infection control measures which are often absent in their training [25]. This is more so critical in the case of Saudi Arabia, where cultural aspects such as hierarchy and the level of management support are key to nurses’ attitudes and self-efficacy. The culture of Saudi society stands to be one of the most critical factors that influence both infection control practices as well as nurse self-efficacy. The profession of nursing has in Saudi Arabia always been shaped by the policies of the state and the culture, which tends to promote or inhibit the process of the professionalization of nurses [26]. For example, Aljohani and colleagues [26] mentioned that nursing education in the Kingdom of Saudi Arabia was largely influenced by foreign factors including American assistance, which may be in contradiction with the cultural values of the area [26]. The large proportion of migrant nurses working in the Saudi medical system may pose additional issues of cultural barriers and communication difficulties [27]. Such factors, in turn, restrict the confidence of the nurses to practice the infection control measures fully and efficiently. The results about self-efficacy in this present study are in alignment with the literature, which positions self-efficacy as one of the determinants that are essential in promoting changes among people in the healthcare setting. In particular, the need to improve measures of self-efficacy in healthcare workers has been stressed, as this could improve their adherence to infection prevention measures [28]. This is especially relevant in Saudi Arabia, where cultural and occupational influences may affect the ability of the nurses to believe that they are capable of performing the required infection prevention measures well.

Findings revealed that male participants exhibited higher attitude scores with respect to infection control than females. However, this study did not manage to find any statistically significant gender differences in knowledge, compliance, and self-efficacy scores. Such observation is congruent with the literature that supports gender as a factor in determining acceptable health practices, particularly infection control practices. For example, Adegboye et al.’s [29] study found male health workers to be more optimistic regarding infection control practices than females, clearly because gender stereotypes and expectancies are socialized in them in terms of their roles in healthcare systems [29]. This socialization could perhaps explain differences in confidence levels, as male nurses are said to be more confident in their clinical skills than female nurses, who may, in turn, enhance their willingness to embrace important infection control processes [30]. Moreover, there is no difference between the male and female respondents in their level of knowledge in this study. This collaborates with findings from other studies that showed no significant differences between males and females concerning knowledge regarding infection control, including standard precautions [31]. This means that educational strategies meant to augment knowledge could be more appropriate for nurses regardless of their gender. On the other hand, complying with infection control measures is related to various factors other than education, such as the type of organizational culture, the level of resources, and even personal belief in adherence to the guidelines [32]. This is particularly pertinent in the Saudi Arabian context, as cultural considerations might further influence the compliance dynamics. Indeed, the vast majority of registered nurses in the Saudi healthcare system are non-Saudi professionals who may have different cultural perceptions that affect infection control attitudes and practices [32]. This cultural intricacy explains the need to appreciate the interplay of gender and cultural aspects in controlling infections among nurses. Lastly, the results concerning self-efficacy among the participants of this study that no differences between male and female nurses also corroborates Bandura’s [7] self-efficacy theory that self-efficacy is said to be more related to experience than gender alone [33]. This strengthens the assumption that all healthcare workers, irrespective of their gender, should have the same level of confidence in their ability to correctly apply infection control procedures. Future educational programs should emphasize enhancing efficacy among nurses through skills development and suitable non-punitive organizational cultures that support compliance with infection control procedures. The relationship between gender, cultural norms, and professional relations is multifaceted, and specific strategies are needed to improve infection prevention and control approaches.

This present study shows that the participants who received training had notably higher knowledge, compliance scores, and general self-efficacy than those who were not trained. This is consistent with other studies that point out the benefit of having organized training structures on the workers’ knowledge and skills in infection control procedures. For example, an earlier study stated that training led to an increase in knowledge of COVID-19 infection control measures among health workers in Saudi Arabia and that it strengthens the idea that organized teaching plans can help understand infection control principles [34]. Moreover, it was pointed out that in order to achieve optimal adherence to infection control measures, there is a need to go beyond mere training to engaging frontline staff through the development of clear messages that will be understood [35]. Although self-efficacy and knowledge have improved, the study observed a discrepancy in attitudinal change between the trained and untrained units. This suggests that while training may increase knowledge and self-efficacy, it does not necessarily change one’s attitude. Earlier studies support this view, noting that the attitudes are determined by a number of factors such as socialization in the healthcare system as well as organizational culture. For instance, it has been indicated that even when there was implementation of infection control practices, the attitudes of the healthcare personnel towards the practices were largely due to systemic issues such as culture and support [36]. With regard to Saudi nationals, cultural issues have a strong bearing on the practices of infection control and nurse’s self-efficiency. The even or dominantly stratified structure of the healthcare system has been reported to influence the perceptions of nurses over their duties and subsequent credibility concerning infection control measures. According to Alsahafi and Cheng [37], communication and cooperation between healthcare workers, which is crucial for achieving effective infection control, may be restricted by cultural standards and reasonable anticipations [37]. Further, the self-efficacy results from this study, which were obtained from the trained group that performed better, are in general accordance with Bandura’s self-efficacy theory which states that self-efficacy is affected not just by gender but by his or her training and experience as well. It strengthens the notion that every member of the healthcare workforce, regardless of their gender, ought to have a commensurate level of faith in their competence to execute infection control procedures. Educational programs in the invitation should aim at enhancing more of nurses’ self-efficacy through providing more true training practices and supportive organizations that motivate reality about control of infection compliance.

Self-efficacy, as well as compliance scores, differ greatly between those who possess the highest educational qualifications. This indicates that education is crucial for professional behavior and self-efficacy in the practice of infection control. This agrees with the work of Aljondi et al. [34], in which staff in Saudi Arabia demonstrated disparate levels of knowledge regarding controls against the spread of COVID-19. The authors, therefore, recommended further education to promote compliance and self-efficacy [34]. Moreover, higher education attainment was related to enhanced self-efficacy and compliance; however, there was no remarkable difference in knowledge and attitude scores. This means that other factors such as training, organizational culture, and experience may have a robust negative impact on knowledge and attitudes towards infection control apart from formal education. Similar observations were made by Rabaan et al. [35] when evaluating the Hajj outbreak of MERS-CoV, pointing out the importance of practical experience and the use of infection control measures [35]. This suggests that while education is important, it should not be relied on to assume how healthcare workers would behave concerning infection control practices. Another perspective that must be taken into consideration is that the infection control practices and the self-efficacy of Saudi nurses are also affected by cultural aspects. The compliance with infection control measures is influenced by culture-specific practices of the people. It has been brought to light by the COVID-19 pandemic that some areas are hard to impose infection control over because of cultural practices, for example, large numbers of people praying in Mecca and Medina [38]. This cultural context calls for the development of more focused training programs that take into consideration local practices and beliefs to make them more efficient in tackling the infection control issue. As such, this only validates that similar initiatives could be implemented, taking into account the many cultural and working circumstances in Saudi Arabia.

The current study revealed interesting findings regarding knowledge, attitude, and general self-efficacy scores as they relate to years of experience. While compliance improved with experience, there were no significant differences in knowledge, attitude, and self-efficacy scores. These results suggest that although the experience may improve compliance, adherence to infection control practice does not change in terms of knowledge attitudinal perception, or self-efficacy. This is in agreement with the findings of Alfahan et al. [39], who noted that training and previous exposure significantly correlate with healthcare professionals’ practices, suggesting that if better practices were looked for, more formal education coupled with specifically tailored training programs rather than years of experiences is the key [39]. Moreover, according to Bandura’s theory of self-efficacy, self-efficacy belief is formed through mastery experience, vicarious experience, and social persuasion [40]. It follows that while experience is a factor in adherence, self-efficacy will be significantly low among trained personnel in the absence of experience or vice versa. Cultural factors emanating from Saudi Arabia also encompass infection control and nurse self-efficacy. In Saudi Arabia, the customs have some impact on the working environment, which, in turn, may have an effect on infection prevention and control adherence. For instance, the religious disposition of healthcare in Saudi Arabia, such as during the Hajj pilgrimage, creates barriers to infection prevention and control. The gaps in health services and inadequacy of culturally appropriate techniques of infection control in Alharbi’s study [41] are very evident, especially after the MERS-CoV outbreak. This concern further necessitates the adjustment of the local rural and customs to make training programs concerning infection intervention meaningful. Study comparisons with practitioners in other countries have appreciable relevance in understanding the situation in Saudi Arabia. For example, research conducted by Iqbal shows that trained healthcare providers are more compliant than untrained ones in this and other regions [42]. Such research strengthens the capacity of other countries to provide appropriate strategies while cautioning them that Saudi Arabia is different in many areas including culture and customs.

This study revealed significant positive correlations between attitudes and the level of knowledge, approaches, and self-efficacy. This means that those with greater amounts of knowledge and more positive attitudes are more likely to be compliant and exhibit self-efficacy in their practice. This finding about knowledge compliance has been observed in several previous investigations, which showed that training and education increase adherence to the stipulated infection control measures. For instance, one study discovered that healthcare personnel who were taught standard precaution measures were more compliant with these measures when compared to those who were not trained [43]. This emphasizes the crucial importance of seeking educational strategies to promote compliance in healthcare environments. Moreover, the positive relationship between self-efficacy and adherence is consistent with Bandura’s social cognitive theory, which states that beliefs of self-efficacy impact the capacity of people to fully accomplish the required activities. In the healthcare field, self-efficacy can be beneficial to nurses as it raises their expectations that they can carry out infection control measures. This was also ascertained by Moxley, who argued for the significance of self-efficacy in teamwork [44]. Thus, the improvement of self-efficacy through training and assistance that targets specific aspects of health management compliance with infection control practices is a priority that needs to be addressed. The cultural context of Saudi Arabia also significantly influences how infection control assessments as well as nurse self-efficacy are executed. Factors of an infection control nature may arise from the sociocultural context of healthcare activities, such as during religious talks. The study by Alharbi et al. [41] reported an alarming gap in culture-focused infection control, especially with the prevalence of COVID-19, where large crowds make it practically impossible to follow laid-down health measures [41]. This cultural context can inform the design of the training programs that aim to improve infection control by targeting local practices and beliefs. Additional studies from other countries highlight some of the peculiar factors that exist in Saudi Arabia. For example, a study that was carried out in Ethiopia concerning the prevention of infection by HIV/AIDS through healthcare workers reported similar findings for Saudi Arabia that training and knowledge enhanced compliance with standard precautions [43]. Conversely, a study performed in Jamaica showed that it was very common for healthcare providers to selectively follow particular infection control policy guidelines, which exemplifies the varying degrees of compliance to policies and procedures from cultural and healthcare perspectives [45]. These comparisons illustrate the necessity of tailoring plans to meet the requirements of the regional setting due to altercations in the cultural situations and policies of the country.

The research found that both knowledge and attitudes assist in enhancing compliance, and they also act through self-efficacy to impact compliance. In particular, knowledge was found to impact compliance positively, significantly, and directly, while attitude—also the second one—was marginally significant. These results imply that people with a lot of knowledge and who hold favorable attitudes are more likely to display compliant behavior. The direct relationship between knowledge and compliance conforms with the findings of other authors who have emphasized the need for education and training in promoting compliance with infection control measures. Previous research has demonstrated that nurses’ level of knowledge greatly affected their adherence to infection prevention and control measures, suggesting the need for continual education as a means of fulfilling this relationship [46]. Likewise, it was explained that self-efficacy is important in the process of acquisition of knowledge to practice noting that well-informed healthcare professionals are expected to exhibit ideal practices of infection control [47].

Moreover, current results outlined that self-efficacy acts as an important mediator between knowledge, attitude, and compliance. The indirect effects analysis showed that knowledge’s indirectly affects compliance through self-efficacy and that attitude also has an indirect effect. From this, it can also be inferred that, without self-efficacy, it is impossible to expect behaviors that are compliant out of knowledge and attitudes possessed by others. This corresponds with the work of Russell et al., who have stated that the compliance of nurses with infection control practices was more determined by the attitudes and perceived self-efficacy of nurses than their actual knowledge about infection control [48]. Therefore, enhancing self-efficacy becomes an important aspect, and not only in how a healthcare practitioner understands and practices infection control measures. Moreover, the cultural factors that are characteristic of Saudi Arabia affect the infection control methods and the confidence of nurses. The communal nature of healthcare practices particularly during religious gatherings raises special concerns for infection control. There are some aspects of the disease that compensate for the easy adoption of violent measures that may be exacerbated by the ongoing COVID-19 pandemic, since great numbers of people might not be able to follow health guidelines [41]. Such a cultural context calls for the formulation of effective and persuasive infection control policies that take into consideration local customs and beliefs. As explained by Liu et al. [49], infection control measures self-efficacy interventions have been shown to increase such measures; hence, organizations can facilitate knowledge translation into practice by boosting nurses’ confidence in doing the work related to infection control.

The total effects analysis revealed a significant total effect of knowledge on compliance indicating that both direct and indirect pathways contribute substantially to compliant behavior. In contrast, the total effect of attitude on compliance was smaller, suggesting that while attitudes are important, their influence is less pronounced than that of knowledge. The findings align with previous research that emphasizes the critical role of knowledge in promoting compliance with infection control protocols. For instance, Huis et al. conducted a systematic review that highlighted the importance of educational interventions in improving hand hygiene compliance among healthcare workers, indicating that knowledge is a key determinant of adherence to infection control measures Mazza et al. [50]. Similarly, it was found that while knowledge is essential, interventions targeting behavioral constructs such as attitudes and self-efficacy may yield more significant improvements in compliance [24]. This underscores the necessity of not only providing information but also fostering positive attitudes and self-efficacy among healthcare professionals.

Findings revealed that self-efficacy has a strong and positive path coefficient on compliance and thus suggests that individuals with high levels of self-efficacy tend to be more compliant. This observation conforms to the findings of Mazza et al. [50]. Accordingly, self-efficacy is important in initiating and sustaining recommended health behavior, particularly during the pandemic regarding compliance with preventive measures [50]. Moreover, the compliance model and the self-efficacy model indicate that the independent variables can explain a substantial portion of the variance in compliance and self-efficacy. This suggests that while knowledge, attitude, and self-efficacy are significant predictors, other factors may also contribute to compliance behaviors. Cultural factors specific to Saudi Arabia, such as the communal nature of healthcare practices and the influence of religious gatherings, may impact how healthcare professionals perceive and implement infection control measures. It was emphasized in an earlier study that there is a need for culturally sensitive approaches to infection control, particularly in light of the challenges posed by large gatherings during the COVID-19 pandemic [41]. To address the gaps identified in this study, it is crucial for nurse managers and educators to implement strategies that enhance self-efficacy. Structured training programs that incorporate mentorship opportunities and routine competency assessments can significantly boost nurses’ confidence in their ability to perform infection control tasks. This aligns with the findings of the previous authors, who noted that self-efficacy-enhancing interventions can lead to improved compliance with health measures [51]. By reinforcing nurses’ confidence, healthcare organizations can facilitate the transfer of knowledge into action, ensuring adherence to essential infection control protocols. The findings of this study highlight the interconnectedness of knowledge, attitude, self-efficacy, and compliance in infection control practices among healthcare professionals in Saudi Arabia. Integrating insights from comparative studies and considering the cultural context, targeted strategies can be developed to enhance infection control practices and nurse self-efficacy, ultimately improving patient safety and healthcare outcomes.

### 4.1. Implication of the Study

This study highlights the importance of self-efficacy in shaping knowledge, attitudes, and, ultimately, adherence to standard precautions among healthcare workers. Nurse educators and hospital managers need to work together in order to provide the means and tools that will undoubtedly enhance the nurses’ self-efficacy and knowledge regarding compliance with standard precautions. Nurse educators must focus on developing and executing well-organized training programs that integrate teaching, practice, and simulation or role-playing methodologies. It is also important to start mentorship programs for younger or novice nurses where experienced nurses can guide them. For nurse educators and the administrative staff, a culture of ongoing training needs to be created by conducting training workshops and conferences. Sharing excellent infection control attitudes can be facilitated by rewarding and celebrating the staff for complying with controls, discussing how to successfully control infections, and promoting free communication. Administrators should implement minimum staffing adequacy, provide compliance with PPE and guidelines, and manage aspects leading to staff exhaustion. Self-efficacy, compliance with infection control practices, and the quality of care and safety of patients can be improved by nurse educators and nurse administrators through addressing these key areas.

### 4.2. Study Limitation

The authors would like to highlight a number of factors that could influence the interpretation of the outcomes. This study is a cross-sectional study and, therefore, by design, we are not able to make causal statements about the relationships between the variables being studied. Therefore, while we are able to note relationships and trends between knowledge, attitude, self-efficacy, and compliance, we cannot state that A changes B at any point during the study. Furthermore, the emphasis on a single region in the Kingdom of Saudi Arabia may also limit the applicability of our findings to other regions which may have different health contexts. Different regions may have different cultural practices, healthcare habits, and/or resource constraints which may impact infection control practices. Therefore, caution should be taken when applying the findings of this study to different populations. Also, self-reporting through questionnaires contributes to another source of potential bias and limitation. Self-reports are susceptible to recall errors, biases toward social approval, and reporting errors which may lessen the reliability of the information collected. Studies show that reliance on self-reporting among sample research participants can introduce bias in findings as the respondents can be untruthful and inaccurate in the information they provide [52]. This limitation is particularly relevant in the context of healthcare behaviors, where individuals who are subject to social pressure tend to exaggerate their adherence to infection prevention and control measures. Lastly, the relatively small sample size of participants in this study raises concerns about the representativeness of the sample. The inadequately small size of the sample limits the statistical power of the findings and does not properly reflect the views and experiences of the healthcare employees [53]. In light of these limitations, further research might benefit from selecting a bigger and wider sample of the population.

## 5. Conclusions

This study confirms the existence of a high association between knowledge and attitudes as well as self-efficacy in determining compliance with standard precautions among healthcare professionals. It is also worth mentioning that the level of compliance increased as the level of knowledge and attitudes became more favorable. In addition, self-efficacy was found to occupy a significant position within this relationship, stressing the importance of this construct in the area of knowledge and positive attitudes. These results suggest a need for improved interventions that can boost the knowledge levels of nurses on infection control and also create a positive attitude towards acquired infection prevention in them and enhance their confidence in regular compliance with standard precautions. Through the effective management of such aspects, nurses’ infection control practices will be enhanced, which will positively reflect on patient safety.

## Figures and Tables

**Table 1:** Demographic characteristics of the participants. N = 305.

Demographics		Frequency	Percent
Gender	Female	233	76.4
	Male	72	23.6
Did you receive training on standard precaution or infection control?	No	15	4.9
	Yes	290	95.1
Highest Degree	Bachelor	161	52.8
	Diploma	85	27.9
	Masters	46	15.1
	Doctorate degree	13	4.3
Years of working Experience	1 year to 5 years	183	60.0
	6 to 10 years	81	26.6
	11 to 15 years	23	7.5
	16 years and above	18	5.9

**Table 2:** Level of knowledge, attitudes, compliance, and general self-efficacy.

Variables	Mean	SD	Interpretation
Knowledge	19.00	2.17	Moderate
Attitudes	55.86	10.22	Moderate
Compliance	72.44	11.47	Intermediate
Self-efficacy	29.99	11.40	Moderate

**Table 3:** Differences in knowledge, attitudes, compliance, and general self-efficacy based on demographic profiles.

**Gender**		**Mean**	***t*-Test**	***p*-Value**	**Remarks**
Total Knowledge Score	Female	18.9957	−0.1096	0.9128	Not Significant
	Male	19.0278			
Total Attitude Score	Female	53.6481	−6.0527	0.0000	Significant
	Male	63.0139			
Total Compliance	Female	72.3906	−0.1334	0.8939	Not Significant
	Male	72.5972			
Total General Self Efficacy	Female	30.2918	0.8306	0.4068	Not Significant
	Male	29.0139			
**Trainings Attended**		**Mean**	***t*-Test**	***p*-Value**	**Remarks**
Total Knowledge Score	Yes	19.2276	2.9016	0.0115	Significant
	No	14.6667			
Total Attitude Score	Yes	55.9345	0.5665	0.5715	Not Significant
	No	54.4000			
Total Compliance	Yes	72.9310	2.3836	0.0311	Significant
	No	62.9333			
Total General Self Efficacy	Yes	30.5828	7.6679	0.0000	Significant
	No	18.5333			
**Highest Degree**		**Mean**	**F-Test**	***p*-Value**	**Remarks**
Total Knowledge Score	Bachelor	19.130	1.9403	0.1231	Not Significant
	Diploma	18.541			
	Masters	19.283			
	Doctorate degree	19.462			
	Total	19.003			
Total Attitude Score	Bachelor	55.149	1.2027	0.3090	Not Significant
	Diploma	57.224			
	Masters	55.022			
	Doctorate degree	58.692			
Total Compliance	Bachelor	73.820	7.7768	0.0001	Significant
	Diploma	68.129			
	Masters	76.630			
	Doctorate degree	68.692			
Total General Self Efficacy	Bachelor	30.745	11.4182	0.0000	Significant
	Diploma	24.906			
	Masters	36.000			
	Doctorate degree	32.615			
**Years of Experience**		**Mean**	**F-Test**	***p*-Value**	**Remarks**
Total Knowledge Score	1 year to 5 years	18.9617	1.4785	0.2204	Not Significant
	6 to 10 years	19.3827			
	11 to 15 years	18.8261			
	16 years and above	17.9444			
Total Attitude Score	1 year to 5 years	56.6885	1.3460	0.2596	Not Significant
	6 to 10 years	55.0123			
	11 to 15 years	52.6957			
	16 years and above	55.2778			
Total Compliance	1 year to 5 years	73.6995	3.1277	0.0261	Significant
	6 to 10 years	71.9630			
	11 to 15 years	67.6522			
	16 years and above	67.8889			
Total General Self Efficacy	1 year to 5 years	30.3279	1.6349	0.1813	Not Significant
	6 to 10 years	30.9877			
	11 to 15 years	27.3478			
	16 years and above	25.4444			

**Table 4:** Correlation matrix between the studied variables.

**Variable**		**Knowledge**	**Attitude**	**Compliance**	**Self-Efficacy**
1. Knowledge	Pearson’s r	-			
	*p*-value	-			
2. Attitude	Pearson’s r	0.256 **	-		
	*p*-value	< 0.001	-		
3. Compliance	Pearson’s r	0.376 **	0.249 **	-	
	*p*-value	< 0.001	< 0.001	-	
4. Self-efficacy	Pearson’s r	0.391 **	0.311 **	0.385 **	-
	*p*-value	< 0.001	< 0.001	< 0.001	-

** Significant at 0.01.

**Table 5:** Standardized path coefficients for the structural equation model.

							**95% Confidence Interval**	
			**Estimate**	**Std. Error**	**z-Value**	***p***	**Lower**	**Upper**
Self-efficacy	→	Compliance	0.254	0.057	4.491	< 0.001	0.143	0.365
Knowledge	→	Compliance	0.115	0.026	4.476	< 0.001	0.065	0.165
Attitude	→	Compliance	0.014	0.007	1.958	0.050	–1.482 × 10^−5^	0.028
Knowledge	→	Self-efficacy	0.154	0.024	6.278	< 0.001	0.106	0.201
Attitude	→	Self-efficacy	0.030	0.007	4.269	< 0.001	0.016	0.044

Note. Delta method standard errors, normal theory confidence intervals, ML estimator.

## Data Availability

The datasets used and/or analyzed during the current study are available from the corresponding author upon reasonable request.
